# 

**Published:** 1903-05

**Authors:** 


### BOOKS RECEIVED.

The Care of the Baby. A Manual for Mothers and Nurses, containing Practical Directions for the Management of Infancy and Childhood in Health and in Disease. By J. P. Crozer Griffith, M. D., Clinical Professor of Diseases of Children in the Hospital of the University of Pennsylvania; Physician to the Children's Hospital, Philadelphia. Third edition, thoroughly revised. 12mo volume of 436 pages, fully illustrated. Philadelphia, New York, London: W. B. Saunders & Co. 1903. [Cloth, $1.50 net.]

Uterine and Tubal Gestation. A Study of the Embedding and Development of the Human Ovum, the early Growth of the Embryo, and the Development of the Synctium and Placental Gland. By Samuel Wyllis Bandler, M. D., Instructor in Gynecology in New York. Post-Graduate Medical School. Small octavo, pp. 170. Illustrated by 93 drawings. New York: William Wood & Company. 1903. [Price, $1.50.]

Diseases of the Heart and Arterial System. Designed to be a Practical Presentation of the Subject for the Use of Students and Practitioners of Medicine. By Robert H. Babcock, A. M., M. D., Professor of Clinical Medicine and Diseases of the Chest, College of Physicians and Surgeons, Medical Department of the Illinois State University, Chicago. Octavo, pp. 874. With three colored plates and 139 illustrations New York and London: D. Appleton & Company. 1903. [Price, $6.00.]

The Practical Medicine Series of Year Books. Comprising ten volumes on the year's progress in Medicine and Surgery. Issued monthly under the general editorial charge of Gustavus P Head, M. D., Professor of Laryngology and Rhinology in the Chicago Post-Graduate Medical School. Volume IV. Gynecology. Edited by Emilius C. Dudley, A. M., M. D., Professor of Gynecology, Northwestern University Medical School, Chicago, and William Healy, A. B., M. D., Instructor in Gynecology, Northwestern University Medical School, Chicago: The Year Book Publishers. 1902. [Price, $1 25; entire series, $7.50.]

Tuberculosis. Recast from Lectures Delivered at Rush Medical College, in affiliation with the University of Chicago. By Norman Bridge, A. M., M. D., Emeritus Professor of Medicine in Rush Medical College; Member of the Association of American Physicians. Duodecimo volume of 302 pages, illustrated. Philadelphia, New York, London: W. B. Saunders & Company. 1903. [Cloth, $1 50 net.]

A Textbook of Legal Medicine and Toxicology. Edited by Frederick Peterson, M. D., Chief of Clinic, Nervous Department of the College of Physicians and Surgeons, New York; and Walter S. Haines, M. D, Professor of Chemistry, Pharmacy, and Toxicology, Rush Medical College, in affiliation with the University of Chicago. Two imperial octavo volumes of about 750 pages each, fully illustrated. Philadelphia, New York, London: W. B. Saunders & Company. 1903. [Per volume: Cloth, $5.00 net; Sheep or Half Morocco, $6.00 net.]

Medical Jurisprudence, Insanity, and Toxicology. By Henry C. Chapman, M. D., Professor of Institutes of Medicine and Medical Jurisprudence in the Jefferson Medical College, Philadelphia. Third edition, thoroughly revised, greatly enlarged, and entirely reset. Handsome 12mo volume of 329 pages, fully illustrated, including four colored plates. Philadelphia, New York, London: W. B. Saunders & Company. 1903. [Cloth, $1.75 net.]

The International Medical Annual. A yearbook of Treatment and Practitioner's Index. Thirty-four contributors, American and Foreign. Twenty-first year. Octavo, pp. 750. New York and Chicago: E. B. Treat & Co. 1903. [Price, $3.00.]

American Edition of Nothnagel's Practice. Diseases of the Pancreas, Diseases of the Suprarenal Capsules, and Diseases of the Liver. By Dr. L. Oser, of Vienna: Dr. E. Neusser, of Vienna; and Drs. H. Quincke and G. Hoppe-Seyler, of Kiel. The entire volume edited, with additions, by Frederick A. Packard, M. D., late Physician to the Pennsylvania and to the Children's Hospitals, Philadelphia; and Reginald H. Fitz, M. D.; Hersey Professor of the Theory and Practice of Physic, Harvard University Medical School, Boston. Handsome octavo of 918 pages, illustrated. Philadelphia, New York, London: W. B. Saunders & Company. 1903. [Cloth, $5.00 net; Half Morocco, $6.00 net.]

American Edition of Nothnagel's Practice. Diseases of the Stomach. By Dr. F. Riegel, of Giessen. Edited, with additions, by Charles G. Stockton, M. D., Professor of Medicine in the University of Buffalo. Handsome octavo volume of 835 pages, illustrated, including 6 full-page plates, Philadelphia, New York, London: W. B. Saunders & Company. 1903. [Cloth, $5.00 net; Half Morocco, $6.00 net.]

Practical Points in Nursing. For Nurses in Private Practice. With an Appendix containing Rules for Feeding the Sick; Recipes for Invalid Food and Beverages; Weights and Measures; Dose List; and a full Glossary of Medical Terms and Nursing Treatment. By Emily A M. Stoney, late Superintendent of the Training School for Nurses, Carney Hospital, South Boston, Mass. Third edition, thoroughly revised. Duodecimo of 458 pages, fully illustrated, including 8 colored and half-tone plates. Philadelphia, New York, London: W. B. Saunders & Company. 1903. [Cloth, $1.75 net.]

